# Anterior Inferior Cerebellar Aneurysm Treated by Aneurysm Resection and Intracranial Artery Anastomosis *in situ*: A Case Report and Literature Review

**DOI:** 10.3389/fsurg.2021.669433

**Published:** 2021-05-25

**Authors:** Chaojue Huang, Shixing Qin, Wei Huang, Yongjia Yu

**Affiliations:** Department of Neurosurgery, First Affiliated Hospital of Guangxi Medical University, Nanning, China

**Keywords:** aneurysms, complex aneurysms, anterior inferior cerebellar aneurysm, aneurysm resection, arterial anastomosis *in situ*

## Abstract

**Background:** Anterior inferior cerebellar artery (AICA) aneurysms are relatively rare in clinical practice, accounting for <1% of all intracranial arteries. After the diagnosis and location are confirmed by angiography, magnetic resonance, and other imaging examinations, interventional, or surgical treatment is often used, but some complex aneurysms require reconstructive surgery.

**Case Description:** An 8-year-old male child was admitted to the hospital due to sudden disturbance of consciousness for 2 weeks. The head CT showed hematocele in the ventricular system with subarachnoid hemorrhage in the basilar cistern and annular cistern. On admission, he was conscious, answered correctly, had a soft neck, limb muscle strength was normal, and had no cranial nerves or nervous system abnormalities. A preoperative examination showed the right side of the anterior distal arteries class under the circular wide neck aneurysm, the distal anterior inferior cerebellar artery supplying a wide range of blood to the cerebellum, the ipsilateral posterior inferior cerebellar artery absent, and the aneurysm close to the VII, VIII nerves. The aneurysm was successfully treated by aneurysm resection and intracranial artery anastomosis *in situ* of a2 AICA-a2 AICA.

**Conclusions:** AICA aneurysms are relatively rare; in this case, a complex wide-necked aneurysm was successfully treated by aneurysm resection and anastomosis *in situ* of a2 AICA-a2 AICA. This case can provide a reference for the surgical treatment of complex anterior cerebellar aneurysms.

## Highlights

- In the treatment of complex aneurysms, bypass reconstruction can effectively ensure the patency of the distal posterior circulation artery and avoid cerebral infarction.
- *In situ* anastomosis technology has good matching of vascular diameter and wall and high expected patency rate, and its patency can be effectively verified by intraoperative fluorescence contrast and ultrasound Doppler.
- It can provide reference for the surgical treatment of complex anterior cerebellar small aneurysms.

## Introduction

Anterior inferior cerebellar artery (AICA) aneurysms are rare clinically, accounting for <1% of all intracranial artery aneurysm. After the diagnosis and location are confirmed by angiography, magnetic resonance, and other imaging examinations, aneurysms are usually treated with intervention embolization or surgery (1–3). However, under the different anatomical locations and morphology of an AICA aneurysm, what kind of treatment is the most effective? There is still no unified conclusion. We report a case of a complex AICA aneurysm. The patient was admitted due to sudden disturbance of consciousness 2 weeks earlier. Physical examination on admission: clear, accurate answers; soft neck; normal muscle strength of limbs; no abnormality in cranial nerve and nervous system examination. CT showed that the ventricular system had hematocele, accompanied by subarachnoid hemorrhage in the basal cistern and annular cistern. Digital subtraction angiography revealed a circular wide-necked aneurysm of the distal AICA with an aneurysm size of about 5.2^*^4.8 mm. The aneurysm was isolated and resected; intracranial anastomosis *in situ* of a2 AICA-a2 AICA was performed. As far as we know, reports of similar cases are rare. Combined with the patient's clinical characteristics, surgical techniques, results, and literature review, the report is as follows:

## Case Description

**Figure 1:**
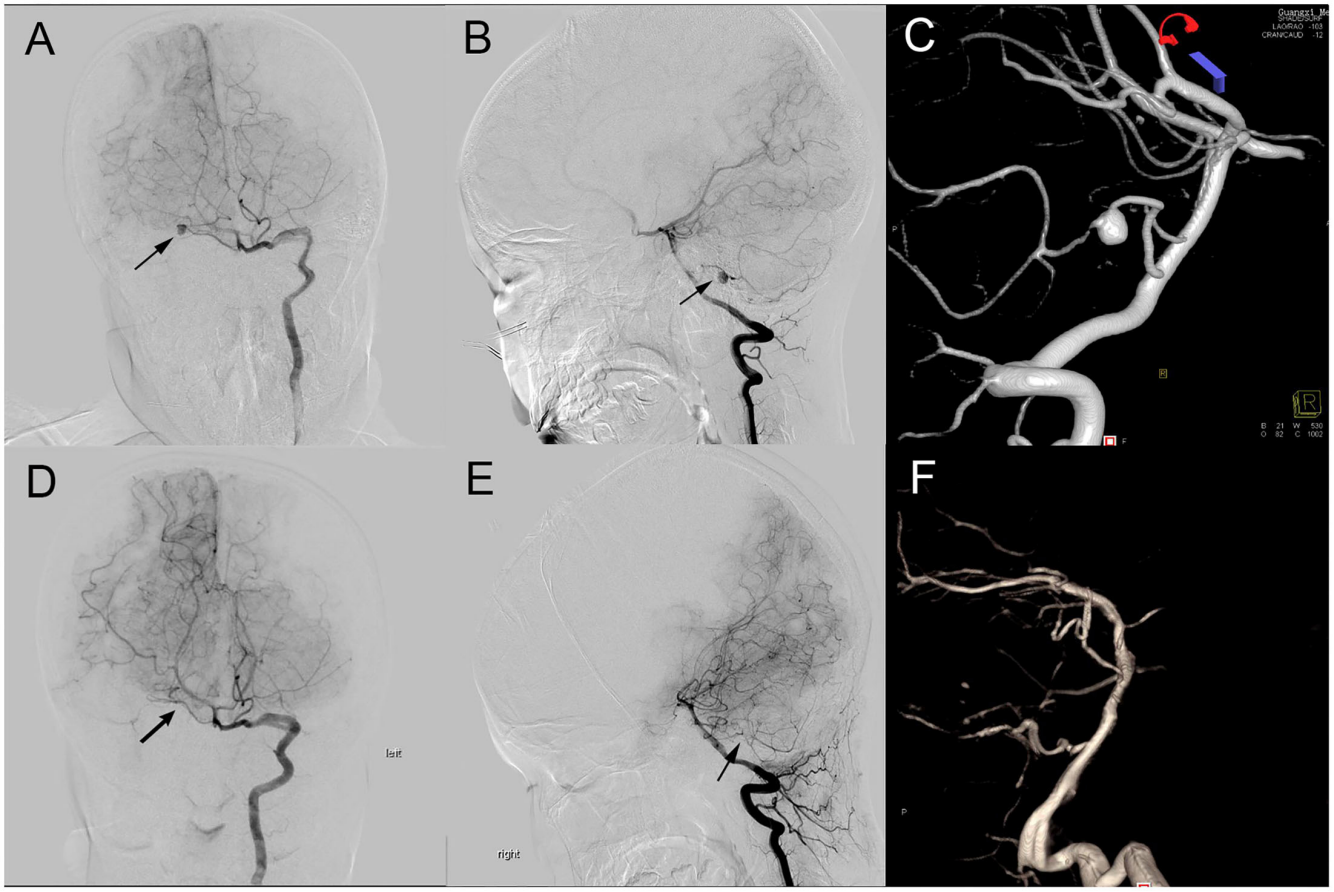
Preoperative and postoperative DSA comparison. **(A–C)**: Preoperative DSA (A, A-P view; B, lateral view) showing an aneurysm (black arrow) located at main trunk of the right AICA; **(D–F)**: Preoperative DSA (D, A-P view; E, lateral view) showing the aneurysm disappeared and the distal AICA patency.

The patient, an 8-year-old male, was admitted December 4, 2020, due to sudden disturbance of consciousness 2 weeks earlier. The patient had a sudden headache and consciousness disturbance 2 weeks earlier, and the head CT showed that the ventricular system had hematocele, accompanied by subarachnoid hemorrhage in the basal cistern and annular cistern. The patient was conscious after symptomatic treatment and then transferred to our hospital for further diagnosis and treatment. Physical examination on admission: clear, accurate answers, soft neck, normal muscle strength of limbs, no abnormality in cranial nerve, and nervous system examination. Digital subtraction angiography revealed a circular wide-necked aneurysm of the distal AICA with an aneurysm size of about 5.2^*^4.8 mm. The distal AICA supplied a wide range of blood, and the ipsilateral posterior inferior cerebellar artery was absent as shown in Figures 1A–C. Magnetic resonance imaging (MRI) shows that the aneurysm was located lateral to the internal auditory canal (IAC), near the VII, VIII nerves. The right AICA was diagnosed, Hunt–Hess class I.

**Figure 2:**
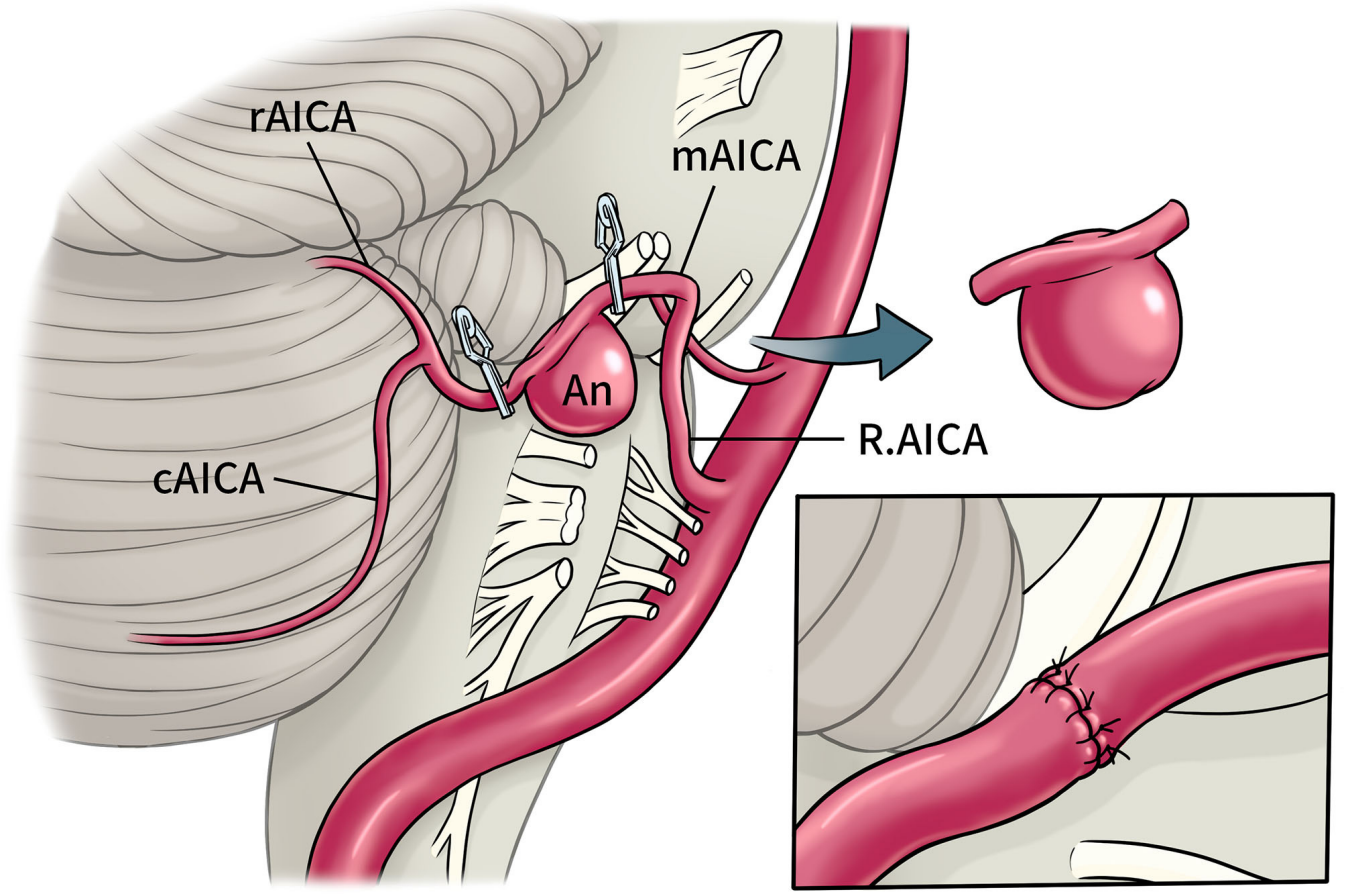
Schematic diagram of operation. Right AICA aneurysm located in a2 segments, intracranial artery anastomosis *in situ* was perform after trapping and resection. (cAICA, caudal trunk of AICA; mAICA, main trunk of AICA; rAICA, rostral trunk of AICA; a2, lateral pontine segments of AICA).

**Figure 3:**
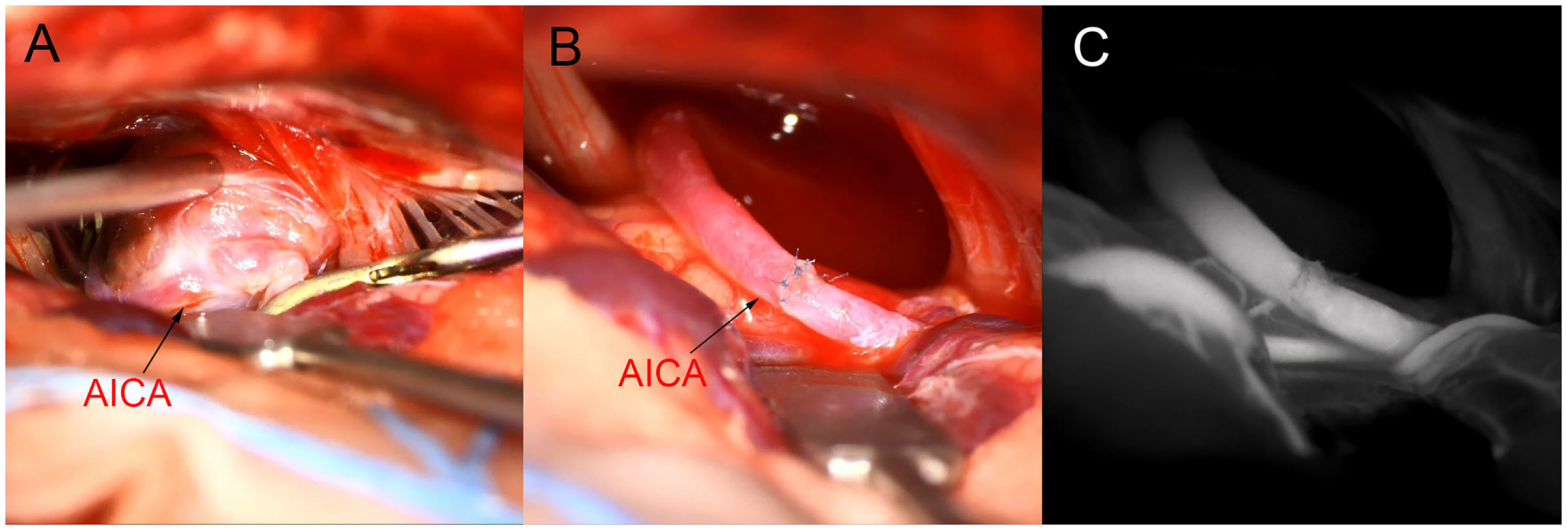
Intraoperative image. **(A)**: Aneurysm neck and parent artery were not clearly defined. **(B,C)**: a2AICA-a2AICA anastomosis *in situ* after aneurysm resection, intraoperative fluorescence imaging confirmed patency.

Craniotomy of the aneurysm by retrosigmoid approach and anastomosis of the AICA was performed under general anesthesia. Intraoperatively, the aneurysm was found to be located in the middle segment of the right AICA, between the facial auditory nerve and the posterior cranial nerve. The aneurysm was closely adhered to the surrounding arachnoid with a wide neck and unclear boundary between the neck and the parent artery (**Figure 3A**). It is estimated that simple clamping is difficult to ensure patency of the parent artery, so we decided to separate the aneurysm from the surrounding adhesion carefully and remove it and part of the parent artery. After dissociating the proximal and distal vessels of the AICA, end-to-end intracranial vascular anastomosis of a2 AICA-a2 AICA was performed *in situ* (the schematic diagram of the operation is shown in Figure 2), and intraoperative fluorescence angiography was performed to confirm the patency of the anastomotic vessels (Figures 3B,C). The patient recovered well after the operation with no facial paralysis, hoarseness, or dysphagia, and limb muscle strength is normal with no ataxia. Cerebrovascular angiography before discharge indicated that the aneurysm disappeared, the distal and proximal ends of the AICA had patency, and the blood supply range was the same as before operation (Figures 1D–F).

## Discussion

AICA aneurysms are relatively rare, and the treatment reports of each center are mainly individual cases and small case series. The total number of aneurysms of the AICA reported by major neurosurgery centers in China accounts for about 0.15–0.3% (1, 2, 4). Symptomatic rupture of the AICA aneurysm is mainly associated with subarachnoid hemorrhage. Some patients show symptoms of mass effect in the cerebellar pontine angle, such as hearing loss, facial paralysis, hoarseness, dysphagia, dizziness, etc., which is related to the location of the aneurysm. Preoperative imaging, the thin layer of magnetic resonance, the bone of the skull base combination CTA reconstruction can effectively locate the aneurysm. AICA aneurysms can be cured by interventional embolization, craniotomy clipping, isolation, and other methods, and the overall effect is satisfied (3, 5–8). A few complex aneurysms require vascular reconstruction (9) and artery anastomosis of the occipital artery. Anterior inferior cerebellar is more common.

According to the relationship between different segments of AICA and the IAC, aneurysm of the AICA can be divided into anterior, internal, and posterior segments of IAC (2). According to the AICA and cerebellar pontine angle region nerve anatomical position relationship, it can be divided into the a1: anterior pontine segments, a2: lateral pontine segments, a3: flocculopeduncular segments, and a4: cortical segments (5, 10). a2 gives rise to the labyrinthine, recurrent perforating, and subarcuate arteries (9). As the a1 segment is deep, and the a4 segment has tiny vessels, a2 and a3 segments can make full use of the space in the CPA region, and the vessels are usually curled and of moderate diameter. They are often used as ideal bypass vessels (9, 11).

When the aneurysm is located in the anterior or internal segment of the IAC, namely segment a1 or a2, interventional embolization is the primary choice of treatment due to the difficulty of anatomical exposure. However, in the case of the tiny AICA, it is difficult to completely ensure the patency of the parent artery during interventional embolization of a complex wide-necked aneurysm, and some patients need to occlude the parent artery (5, 12). Due to the existence of potential perforating arteries, occluding the AICA may lead to facial paralysis, dysphagia, water choking, occipital lobe infarction, binocular hemianopia, etc. (2).

When the aneurysm is located in the posterior segments of IAC, namely segment a3 or a4, application of a retrosigmoid sinus approach can obtain effective surgical anatomy of the exposure; previous literature has reported that the clipping effect is good (1, 4, 13). However, there are also some cases due to the aneurysm shape being broad neck or fusiform, which are difficult to clip. Once the parent artery is narrowed or the aneurysm has a residual neck after clipping, it leads to an increased risk of rebleeding, and these cases can only choose the aneurysm isolation technique. However, when the ipsilateral PICA and SCA is hypoplasia or tiny, when AICA is the mutation of PICA, or when you cannot be sure that variations in the course of the internal auditory artery (IAA) arise from the distal AICA, pure isolated aneurysm or occlusion of the anterior artery can produce a wide range of cerebral infarction and severe neurological dysfunction.

Therefore, for these complex AICAs, the application of the bypass vessel reconstruction technique is an effective strategy to preserve the distal vessel patency and avoid cerebral infarction. This paper reviews and summarizes previous literature reports on vascular reconstruction for complex AICA aneurysms, and most of them achieve good efficacy as shown in Table 1. Pasler et al. (16) and Baranoski et al. (9), respectively, report one case of complex AICA resection by the retrosigmoid sinus approach, followed by end-to-end anastomosis of AICA *in situ*, which effectively avoided postoperative cerebral infarction. Baranoski et al. (9) and Lee et al. (11) also, respectively, report that the p3 segment of PICA was anastomotic with the a3 segment of AICA to isolate proximal aneurysms of AICA and effectively avoid cerebral infarction. No matter which type of vascular reconstruction, this requires the surgeon to be proficient in deep vascular anastomosis. Comparatively, on the one hand, the distal anastomosis between the OA and AICA is superficial but usually requires the application of an incision flap similar to the distal lateral approach. Besides this, there is a risk that the diameter and wall of bypass vessels will not match very well, which affects the anastomosis effect. On the other hand, the anastomosis of PICA-AICA must first be anatomically similar to the branch vessels, and the additional intraoperative risk of blocking PICA may lead to more extensive cerebral infarction after surgery. Compared with the above two vascular reconstruction techniques, end-to-end anastomosis of AICA *in situ* for craniotomy using a retrosigmoid sinus approach can achieve good matching of vascular diameter and wall of bypass vessels, recovery of original hemodynamics, closer to natural anatomy, and high expected patency rate.

**Table 1:** Literature review of AICA aneurysms treated by vascular reconstruction.

**No**.	**Year**	**Author**	**Age/sex**	**Onset**	**Primary**	**Location**	**Size (mm)**	**Types**	**Operation**	**Outcome**
1	2009	Fukushima et al. (14)	15/F	N/A	Yes	a2	14*10.8	Dissecting	OA-AICA bypass	DSA not prompt vascular patency, then interventional embolism aneurysms
2	2010	Oyama et al. (15)	65/F	ICH	Yes	a2	N/A	Fusiform	OA-AICA bypass+trapping+thrombectomy	Legacy swallowing dysfunction, left limbs hemiplegia and gait disorder
3	2011	Päsler et al. (16)	22/M	N/A	Yes	a2	N/A	Globular	Excising the aneurysmal segment, reanastomosis: a2 AICA-a2 AICA	Hearing impairment and headache recurrence 1 year later, MR indicate vascular patency
4	2012	Fujimura et al. (17)	77/F	SAH	Yes	a2, cAICA	N/A	Fusiform	OA-AICA bypass+trapping	Hearing impairment, no infarction, DSA prompt patency.
5	2016	Kanamori et al. (18)	62/M	SAH	Yes	a3, AICA-PICA variant	13	Saccular	OA-AICA bypass+trapping	Right hemiplegia improved, but right abducens nerve palsy did not improve, DSA prompt patency
6	2018	Lee et al. (11)	59/M	N/A	Yes	a2, cAICA	7	Irregular	*In situ* bypass: p3PICA-a3AICA, trapping+thrombectomy	Without neurologic deficits after 6 weeks follow-up, DSA prompt patency
7	2018	Umekawa et al. (19)	78/M	N/A	No	a2, mAICA	5.6	pseudo	OA-AICA bypass+trapping	DSA prompt patency, mRS = 1
8	2020	Hou et al. (20)	53/F	SAH	Yes	a2, cAICA	N/A	dissecting	Excising the aneurysmal, *In situ* suturing: a2AICA-a2AICA	The mild facial paralysis on the right side improved, DSA prompt patency 9 months later
9	2020	Baranoski et al. (9)	75/F	N/A	Yes	a1, mAICA	N/A	fusiform	*In situ* bypass: p3PICA-a3AICA, trapping+thrombectomy	Symptoms improved, after 6 months later, mRS = 0
10	2020	Baranoski et al. (9)	51/F	SAH	No	a2, mAICA	N/A	fusiform	Excising the aneurysmal segment, reanastomosis: a2 AICA-a2 AICA	Recover well, after 1 year follow-up, mRS = 1

*AICA, anterior inferior cerebellar artery; PICA, posterior inferior cerebellar artery; F, female; M, male; mRS, modified Rankin Scale; N/A, not available; OA, occipital artery; SAH, subarachnoid hemorrhage; cAICA, caudal trunk of AICA; mAICA, main trunk of AICA; rAICA, rostral trunk of AICA; a2, lateral pontine segments of AICA; a3, flocculopeduncular segments of AICA; p3, tonsillomedullary segments of PICA*.

## Conclusions

In this case, preoperative angiography suggested that the right PICA was absent, and the blood supply range of the right AICA was extensive. If aneurysm isolation was performed alone, the risk of postoperative cerebellar and brainstem infarction was high. Therefore, the aneurysm was treated by excision of the aneurysm and anastomosis *in situ* of AICA. Intraoperative fluorescence angiography and Doppler imaging confirmed that the vessel patency. Postoperative angiography also indicated that anastomosis patency, and the blood supply range of the anterior cerebellar artery was the same as before. There are few reports on the application of vascular reconstruction in the treatment of AICA aneurysms. This case can provide a reference for the surgical treatment of complex AICA aneurysms.

## Data Availability Statement

The original contributions presented in the study are included in the article/supplementary material, further inquiries can be directed to the corresponding author/s.

## Author Contributions

CH and WH: conceptualization and study design. CH and SQ: data collection, literature research, and manuscript drafting. WH and YY: revision. CH: funding. All authors contributed to the article and approved the submitted version.

## Conflict of Interest

The authors declare that the research was conducted in the absence of any commercial or financial relationships that could be construed as a potential conflict of interest.

## Abbreviations

CT: computed tomography
DSA: digital subtraction angiography
MRI: Magnetic resonance imaging
AICA: Anterior Inferior Cerebellar Artery
IAC: internal auditory canal
PICA: posterior inferior cerebellar artery
SCA: superior cerebellar artery
IAA: internal auditory artery
OA: occipital artery.
